# Correction: Exogenous salicylic acid-induced drought stress tolerance in wheat (*Triticum aestivum* L.) grown under hydroponic culture

**DOI:** 10.1371/journal.pone.0270729

**Published:** 2022-06-24

**Authors:** The PLOS ONE Staff

There is an error in affiliation 5 for author Ali Raza. The correct affiliation 5 is: Key Laboratory of Ministry of Education for Genetics, Breeding and Multiple Utilization of Crops, Oil Crops Research Institute, Center of Legume Crop Genetics and Systems Biology/College of Agriculture, Fujian Agriculture and Forestry University (FAFU), Fuzhou, China.

Additionally, Table 3, “Influence of foliar application of salicylic acid on morphological attributes of wheat cultivars grown under drought stress conditions,” does not appear, and the image for Table 4 appears twice. The table that appears as Table 3 should be the Table 3 provided here in this document. The table captions appear in the correct order. However, the table footnotes will need to be updated. Please view Table 3 here:

**Table 3:** Influence of foliar application of salicylic acid on morphological attributes of wheat cultivars grown under drought stress conditions.

Treatments			Root length (cm)	Shoot length (cm)	Root fresh weight (g)	Shoot fresh weight (g)	Root dry weight (g)	Shoot dry weight (g)
Varieties	Drought stress	Salicylic acid (mg L^-1^)±SE						
` **Barani-17**	**D**_**0**_	**S**_**0**_	38.67 l	56.33 c	8.11 c	14.57 de	1.22 d	1.39 c
		**S**_**1**_	40.67 l	62.67 b	9.35 b	16.50 bc	1.37 c	1.74 b
		**S**_**2**_	43.67 k	65.67 a	9.63 b	17.53 ab	1.71 c	1.78 b
		**S**_**3**_	44.00 k	68.00 a	11.15 a	18.77 a	1.81 a	1.99 a
	**D**_**1**_	**S**_**0**_	52.33 f-I	52.00 ef	6.33 e-h	10.44 hi	1.02 i	1.02 ij
		**S**_**1**_	54.67 d-f	53.67 de	7.47 c-e	11.60 gh	1.13 e-g	1.22 f
		**S**_**2**_	52.33 f-i	54.67 cd	7.37 c-e	12.50 fg	1.15 ef	1.25 ef
		**S**_**3**_	51.00 ij	55.33 cd	7.68 cd	12.67 fg	1.16 e	1.25 ef
	**D**_**2**_	**S**_**0**_	54.33 d-g	48.00 h	4.70 i-l	8.60 j	0.90 j	1.00 ij
		**S**_**1**_	53.00 e-i	49.00 gh	5.30 h-k	9.50 ij	1.02 i	1.05 hi
		**S**_**2**_	52.00 g-i	51.00 fg	5.37 h-j	9.57 ij	1.09 gh	1.10 gh
		**S**_**3**_	55.00 de	53.00 d-f	5.77 g-i	11.44 gh	1.10 fg	1.12 g
**Anaj-17**	**D**_**0**_	**S**_**0**_	58.00 ab	42.67 jk	5.78 g-i	11.44 gh	0.83 k-m	1.22 f
		**S**_**1**_	55.67 b-d	44.67 ij	6.63 d-g	12.67 fg	1.02 i	1.24 ef
		**S**_**2**_	53.67 d-h	46.67 hi	6.14 f-h	12.60 fg	1.04 hi	1.28 de
		**S**_**3**_	55.33 c-e	47.67 h	7.17 c-f	15.53 cd	1.11 e-g	1.31 d
	**D**_**1**_	**S**_**0**_	51.67 hi	33.67 m	4.28 j-m	8.61 j	0.81 m	0.82 m
		**S**_**1**_	55.00 de	39.67 l	4.77 i-l	10.21 hi	0.86 j-l	0.91 kl
		**S**_**2**_	57.67 a-c	41.67 kl	5.51 g-i	10.43 hi	0.88 jk	0.96 jk
		**S**_**3**_	54.67 d-f	42.67 jk	5.67 g-i	13.50 ef	0.91 j	0.98 j
	**D**_**2**_	**S**_**0**_	59.67 a	28.67 o	3.14 m	6.65 k	0.61 n	0.73 n
		**S**_**1**_	55.00 de	30.67 no	3.67 lm	9.14 ij	0.81 m	0.85 m
		**S**_**2**_	53.00 e-i	32.67 mn	3.70 lm	10.44 hi	0.82 lm	0.86 lm
		**S**_**3**_	49.00 j	34.00 m	4.18 k-m	11.67 gh	0.84 k-m	0.87 lm
**C**			555.56 **	5202.00**	95.15**	54.06**	2.12**	1.89**
**DS**			205.29**	1086.76**	74.25**	178.83**	0.87**	2.02**
**SA**			3.20**	145.67**	7.22**	46.16**	0.23**	0.17**
**C×DS**			318.35**	29.63**	3.55**	19.87**	0.15**	0.08**
**C×SA**			18.85**	0.33ns	0.24ns	2.18**	0.02**	0.03**
**DS×SA**			21.55**	4.32**	0.49**	0.15ns	0.03**	0.01**
**C×DS×SA**			26.64**	11.40*	0.40*	1.85**	0.02**	0.02**

*Significant at 0.05 level of significance;

**Significant at 0.01 level; ns, non-significant; C, cultivar; DS, drought stress; SA, salicylic acid; Means not sharing the common letter differ significantly.

The publisher apologizes for the errors.
